# Burial, Funerals and Mourning

**Published:** 1912-10

**Authors:** 


### EDITORIAL DEPARTMENT.

DR. F. E. DANIEL, Editor DR. R. H. L. BIBB, Associate Editor

DEPARTMENTS { EUGENICS: Drs. M. Duggan and T. Y. Hull.

WOMAN'S DEPARTMENT: Mrs. F. E. Daniel.

### Burial, Funerals and Mourning.

Inhumation was first instituted and practiced by the Jews. In early ages and amongst other ancient peoples the more rational method of disposing of the human dead was incineration. The ostensible object of burial is to get rid of the body,—just that much dead organic matter; to enable the microbes in the earth to convert it as rapidly as may be into its original inorganic elements; and yet, with that inconsistency which seems to characterize so many of our affairs, we do all in our power to defeat this object. The corpse is embalmed, to prevent decay; and it is enclosed in an air-tight metal casket, thus rendering resolution into the inorganic impossible. It is time that the silly sentiment which attaches to the dead body be done away with. It is a survival of that foolish custom practiced by the Peter-the-Hermit-Crusaders of bringing back the bones of those who fell; and every enlightened community should have a furnace for cremating the dead.

It is astonishing with what tenacity people hold to customs hoary with age, and persist in doing that which is irrational and out of date. The funeral procession, a large crowd of supposed mourners following a plumed funeral car is the remains of the medieval custom of carrying the plumed helmet of a fallen knight at the head of a procession of his followers, by his esquire, who leads the favorite war-steed, laden with the knight's arms and accoutrements. We imitate it to this day, and every dead body is followed to the grave by everybody, mourners or no, and the crowd stands bareheaded in the sun, or the wind or rain, while a long-winded preacher drawls out a lot of solemn stuff, all looking solemn; whereas, doubtless, in many cases, the death of the party is a blessed relief to the family, and a cause, rather, for rejoicing; e. g., a drunken, brutal husband and father.

Yet the family must put on black, *cap-a-pie*. It is the custom. It is a survival of the Jews' sackcloth and ashes. They must go to an expense they often can not afford, and put on "mourning," which is unhygienic, unbecoming, unnecessary, irrational and often a lie and conceals a hypocrite. It is a bid for sympathy, not needed; it says to the world: "Behold how grieved I am; aren't you sorry for me?"

It is time the medical profession should insist upon reform in these matters and preach a crusade against the public funeral, the embalming of bodies and the wearing of mourning. Funerals should be private. Let friends meet at the home of the deceased and hold such ceremonies as the family desires, and with doors and windows open and God's blessed sunshine streaming in the room, amidst flowers, and perhaps to the accompaniment of bird song without, and then the remains should be taken charge of by officers of the law, the undertaker, and the sexton, to be decently disposed of. Let no black crepe be tacked at the door. A funeral, to many families, is a calamity, indeed. It is "customary" to buy an expensive metallic casket, and hire a lot of carriages for Tom, Dick and Harry, to whom it is sometimes a pleasant outing at the widow's expense, who really can't afford it. This is all wrong, and should be done away with. In addition to all this useless expense is the purchase of a lot, and the erection of a grave stone. The lots are, as a general thing, held by tight speculators, who know that one just has to have it, and makes his price accordingly. Away with embalming, metal caskets, inhumation, funeral processions—and "mourning." This is said to be an age of progress, but we progress in everything except in theology and the disposal of the dead.

